# Reduced Expression of PD-1 in Circulating CD4+ and CD8+ Tregs Is an Early Feature of RRMS

**DOI:** 10.3390/ijms23063185

**Published:** 2022-03-16

**Authors:** Maja Machcińska, Magdalena Kierasińska, Martyna Michniowska, Marta Maruszewska-Cheruiyot, Ludmiła Szewczak, Rafał Rola, Anna Karlińska, Michael Stear, Katarzyna Donskow-Łysoniewska

**Affiliations:** 1 Laboratory of Parasitology, General Karol Kaczkowski Military Institute of Hygiene and Epidemiology, 01-163 Warsaw, Poland; maja.machcinska@gmail.com (M.M.); magdalena.glaczynska@wihe.pl (M.K.); marta.maruszewska@wihe.pl (M.M.-C.); ludmila.szewczak@wihe.pl (L.S.); 2 Department of Parasitology, Institute of Functional Biology and Ecology, Faculty of Biology, University of Warsaw, 00-096 Warsaw, Poland; martyna.michniowska@gmail.com; 3 Department of Neurology, Military Institute of Aviation Medicine, 01-755 Warsaw, Poland; rrola@wiml.waw.pl (R.R.); a.karlinska@wiml.pl (A.K.); 4 Department of Animal, Plant and Soil Science, Agribio, La Trobe University, Bundoora, Melbourne, VIC 3086, Australia; m.stear@latrobe.edu.au

**Keywords:** T cells, suppressive markers, cytokines, relapsing–remitting multiple sclerosis

## Abstract

Altered regulatory T cell (Treg) function could contribute to MS. The expression of activating and inhibitory receptors influences the activity of Tregs. Our aim was to investigate T cell phenotypes in relapsing–remitting MS (RRMS) patients at an early phase of the disease. We examined the influence of demographic parameters on the distribution of CD4+ and CD8+ T cell subclasses by generalized linear modeling. We also studied the expression of the following markers—CTLA-4, GITR, PD-1, FoxP3, Helios, CD28, CD62L, CD103—on T cell subsets from peripheral blood with a 14-color flow cytometry panel. We used an antibody array to define the profiles of 34 Th1/Th2/Th17 cytokines in the serum. Expression of PD-1 and GITR on CD4+ and CD8+ Tregs was decreased in RRMS patients. The proinflammatory factors IFN-*γ*, IL-17, IL-17F, TGF*β*-1, TGF*β*-3, IL-1SRII, IL-12 p40, sgp130, IL-6sR were significantly increased in RRMS patients. Therefore, a deficiency of PD-1 and GITR immune checkpoints on CD4+ and CD8+ Tregs is a feature of RRMS and might underlie impaired T cell control.

## 1. Introduction

Multiple sclerosis (MS) is a disease with continuous inflammatory activity within the CNS, giving rise to tissue damage that results in brain and spinal cord atrophy and irreversible neurological disability. However, the pathogenesis remains elusive. This poses a particular challenge given that MS is a serious neurological disorder that affects mainly young Caucasians, especially women, usually with an age of onset at 18 to 40 years. Among them, relapsing–remitting (RRMS) is the most frequent clinical form of MS, affecting approximately 80% to 85% of people with MS.

CD4+ T lymphocytes have been widely studied and their role in autoimmune diseases is well recognized [1,2]. However, mounting evidence suggests that CD8+ T cells play an important role in protection against autoimmune diseases [3,4,5,6]. The CD4 and CD8 subpopulations of Tregs determine the regulatory balance. Functionally defective Tregs contribute to the pathogenesis of MS [7,8,9]. Recent studies in MS point to a change in immune dynamics during disease relapse periods that is strongly connected with the influence of inflammatory cytokines on CD8+ T cells. Acute MS relapses are characterized by a substantial deficit in the suppressive ability of CD8+ T cells [10].

CD8+ regulatory T cells are a highly suppressive subtype of CD8+ T cells, characterized by their ability to suppress the activation and proliferation of autoreactive CD4+ effector cells by cell-to-cell contact [11]. The inhibitory cell-surface receptors act as immune checkpoints, serve as a second activation signal for T cells and form a network of positive and negative signals that fine tune immune responses to infection while restricting immunity towards the self. These proteins offer a promising strategy for treating autoimmune diseases. 

The cytokine milieu can influence the number of Tregs. Changes in the expression of suppressive markers of T cells in blood and CNS lesions reflect altered numbers and activity of Tregs. Changes in the expression of suppressive markers could also influence cytokine synthesis [12,13]. Therefore, the analysis of activating and inhibitory receptors on T cell subsets may contribute to a better understanding of MS pathogenesis. 

Here, we designed 14-color flow cytometry panels for peripheral blood T cells and defined markers for immunomodulatory cell-surface receptors and transcription factors on regulatory CD4+ and CD8+ T cells. We characterized subsets of suppressive CD8+ T cells (CD8+CD122+Helios+, CD8+CD25+FoxP3+) that are altered in the common clinical forms of untreated relapsing–remitting MS (RRMS) patients, with active episodes of neurological deficit (relapses), and analyzed the associations between these subsets and demographic parameters. We also described the cytokine profile in serum. 

## 2. Results

### 2.1. Characteristics of the Study Population

All patients and controls were ethnically Caucasian but there was a higher proportion of females in the RRMS patients (13/15) than in the healthy controls (12/35). The RRMS patients were, on average, younger, with a median age of 30 years, compared to a median age of 38.5 years in the controls. These differences mandated the use of generalized linear models that adjusted for differences between sex and age.

### 2.2. Demographic Factors Affect the Treg Populations

**Figure 1:**
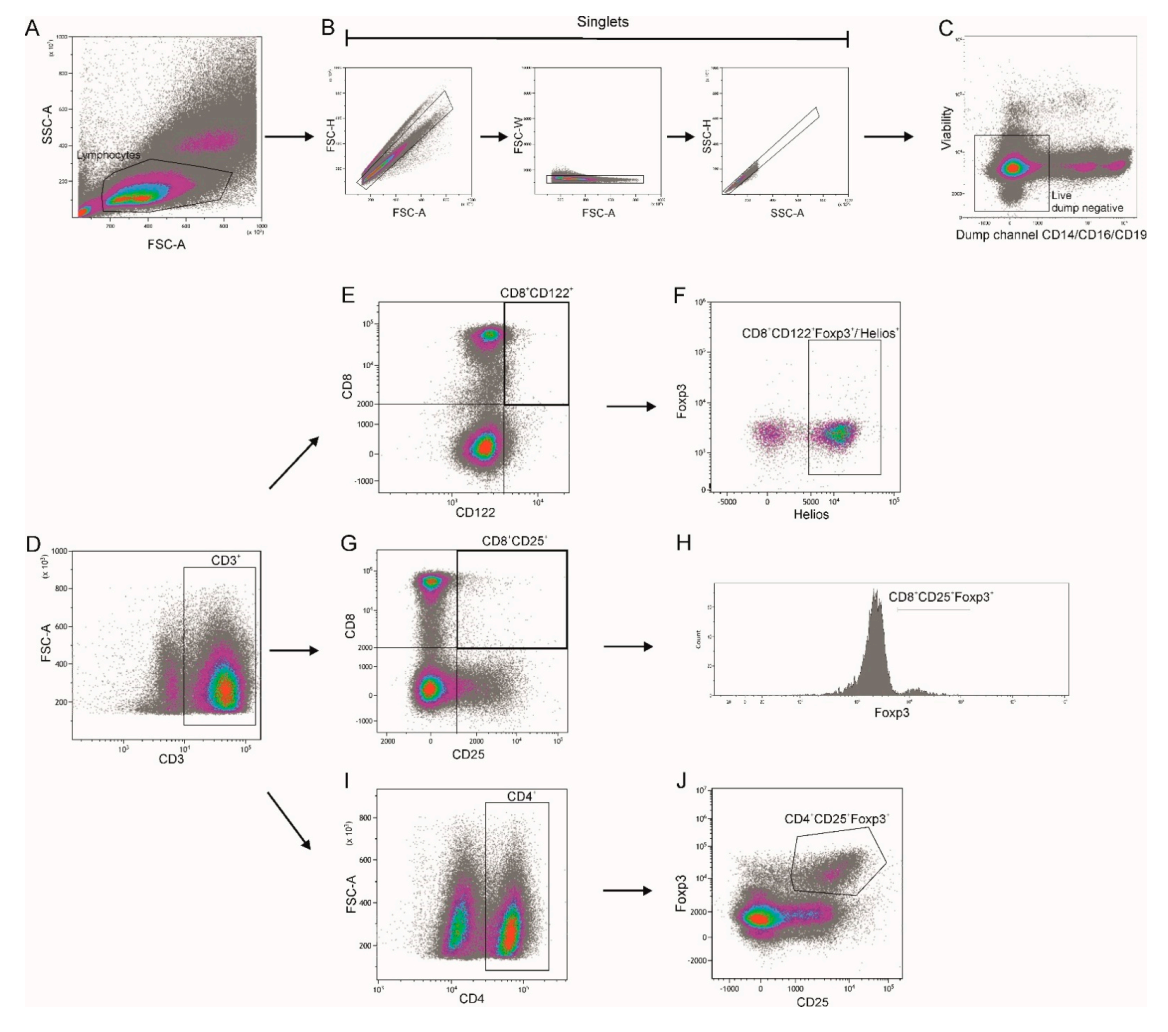
Gating strategy for CD4+ and CD8+ Treg populations. Gating strategy for flow cytometric analysis of lymphocyte population to identify CD4+CD25+FoxP3+, CD8+CD25+FoxP3+ and CD8+CD122+Helios+ Tregs. A time gate was initially applied to exclude any electronic noise and artifacts (not shown here). Next, based on size and granularity, lymphocytes were gated in a forward scatter area (FSC-A) versus side scatter area (SSC-A) plot (**A**). Then, doublet cells were excluded using FSC-A/FSC-height (FSC-H), FSC-A/FSC-Width (FSC-W) and SSC-A/SSC-height (SSC-H) parameters (**B**). Next, viable lymphocytes were gated and cells that expressed monocyte, NK cell and B cell lineage markers CD14, CD16 and CD19, respectively were excluded (“dump channel”, dump negative gate) (**C**), followed by expression of CD3 (**D**). CD8+CD122+Helios+ Tregs were identified as CD8+CD122+ cells (**E**) with FoxP3^+/−^ and Helios co-expression (**F**). CD8+CD25+FoxP3+ Tregs were identified by CD8 and CD25 (**G**) and Foxp3 (**H**) expression. CD4+CD25+FoxP3+ Tregs were identified as CD4^+^CD25^+^FoxP3^+^ cells (**I**,**J**). Representative plots are presented.

Using flow cytometry analyses, three major subsets of T cells were defined: CD4+CD25+, CD8+CD122+ and CD8+CD25+. Each subset had a distinct expression of the intracellular markers: forkhead box P3 (FoxP3) and Helios (Ikzf2) transcription factor. Based on the expression of FoxP3 and Helios, CD4+CD25+FoxP3+, CD8+CD25+FoxP3+ and CD8+CD122+Helios+ (defined as CD8+CD122+FoxP3+/-Helios+) T cells were identified. Identification of Treg populations was based on the gating strategy presented in Figure 1.

The mean percentage of CD8+CD122+ cells was 2.77 ± 0.42 (mean ± sem). The distribution of CD8+CD122+ cells among individuals was right-skewed and not significantly different from a gamma or a log-normal distribution. Patients had a lower percentage of CD8+CD122+ cells (1.07 ± 0.59) than controls (3.50 ± 0.48; *p* = 0.007) but there were no significant effects of gender (*p* = 0.604) or age (*p* = 0.473). Further analysis within patients showed that there was no association between the Kurtzke Expanded Disability Status Scale (EDSS) and the percentage of CD8+CD122+ cells (*p* = 0.694). Within patients, gender (*p* = 0.954) and age (*p* = 0.992) were not associated with the percentage of CD8+CD122+ cells (Table 1).

**Figure 2:**
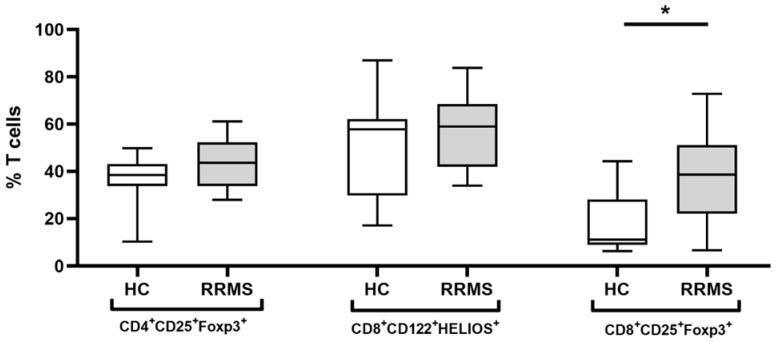
Frequency of CD4+ and CD8+ Treg populations. Comparison of the frequencies of CD4+CD25+FoxP3+ (among CD4+CD25+), CD8+CD25+FoxP3+ (among CD8+CD25+) and CD8+CD122+Helios+ (among CD8+CD122+) Treg populations in healthy controls (HC) and RRMS patients. * *p* ≤ 0.05.

The mean percentage of CD8+CD122+Helios+ cells was 53.1 ± 3.15. The distribution was slightly left-skewed but not significantly different from a normal distribution. There was no significant difference between patients and controls in the percentage of CD8+CD122+Helios+ cells (*p* = 0.211); similarly, there was no detectable effect of age (*p* = 0.973) but there was a higher percentage of CD8+CD122+Helios+ cells in females (64.5 ± 4.0) than males (43.9 ± 4.7; *p* = 0.005). Within patients, the number of CD8+CD122+Helios+ cells was not affected by the EDSS (*p* = 0.381) or by age (*p* = 0.887) but there was an effect of gender (*p* = 0.006). The mean in females was 63.7 ± 3.9 while the mean value in males was only 45.9 ± 4.2 (Table 1, Figure 2).

The mean percentage of CD8+CD25+ cells was only 0.45 ± 0.09. The distribution was right-skewed and not significantly different from a gamma or a log-normal distribution. There were no significant differences due to disease status (*p* = 0.412), age (*p* = 0.119) or gender (*p* = 0.854). Within patients, the EDSS (*p* = 0.093), age (*p* = 0.102) and gender (*p* = 0.818) were not significantly associated with differences in the number of CD8+CD25+ cells (Table 1).

The mean percentage of CD8+CD25+FoxP3+ cells was 24.4 ± 2.93. The distribution was strongly right-skewed and significantly different from the normal (Kolmogorov–Smirnov: *p* < 0.010) and gamma distributions (*p* = 0.016). The log-normal distribution provided a slightly better description (Kolmogorov–Smirnov: *p* = 0.064), although both Anderson–Darling and Cramer von Mises tests had probabilities below 5% (*p* < 0.005 and *p* = 0.006), indicating that statistical inferences need to be made cautiously. Patients had a higher percentage of CD8+CD25+FoxP3+ cells (after back transformation 27.1 ± 1.20) than controls (11.9 ± 1.15; *p* = 0.003) but there was no significant effect of gender (*p* = 0.066) or age (*p* = 0.843). Within patients, gender (*p* < 0.001), but not EDSS (*p* = 0.520) or age (*p* = 0.187), was significantly associated with differences in the percentage of CD8+CD25+FoxP3+ cells. After accounting for sex and age in the generalized linear model, and back transformation, the mean value in females was 37.3 ± 1.21 while the mean value in males was 12.4 ± 1.58 (Table 1, Figure 2). 

The mean percentage of CD4+CD25+ cells was 9.4 ± 0.70. The distribution was slightly right-skewed and bimodal with peaks around 6 and 12 percent. The data were not significantly different from the normal, log-normal or gamma distributions. The *p* values of the EDF tests were higher for the gamma distribution than the other two distributions, and the gamma distribution was used for the generalized linear modeling. Patients had a lower percentage of CD4+CD25+ cells (*p* = 0.023). There was no effect of gender (*p* = 0.832) but older individuals had more CD4+CD25+ cells (*p* = 0.048). After back transformation, the mean percentages in patients and controls were 8.0 ± 1.1 and 11.1 ± 1.1. Among patients, the percentage of CD4+CD25+ cells was influenced by age (*p* = 0.013) and gender (*p* = 0.028) but not EDSS (*p* = 0.144) (Table 1).

The mean percentage of CD4+CD25+FoxP3+ cells was 39.9 ± 1.7. The distribution was slightly left-skewed but not significantly different from a normal distribution. There was no significant effect of disease status (*p* = 0.126), gender (*p* = 0.319) or age (*p* = 0.098). Among patients, EDSS (*p* = 0.882), gender (*p* = 0.910) and age (*p* = 0.338) had no significant effect on the number of CD4+CD25+FoxP3+ cells (Table 1, Figure 2). 

### 2.3. Expression of Immunomodulatory Cell-Surface Receptors and Transcriptional Factors on CD4+ and CD8+ Tregs

**Figure 3:**
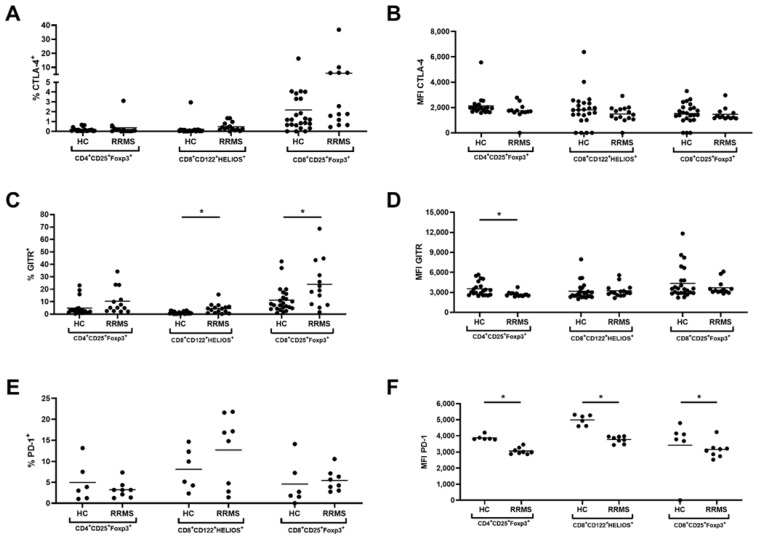
Expression of suppressive markers on CD4+ and CD8+ Treg populations. Comparison of the percentage and expression (MFI) of CTLA-4+ (**A**,**B**), GITR+ (**C**,**D**) and PD-1+ (**E**,**F**) cells within CD4+CD25+FoxP3+, CD8+CD25+FoxP3+ and CD8+CD122+Helios+ Treg populations in healthy controls (HC) and RRMS patients. * *p* ≤ 0.05.

The expression of several cell surface markers specific for T cell identification and function, especially Tregs, was determined. No significant differences were found in the frequency of CTLA-4+ cells, apart from the significantly higher percentage of CD8+CD122+ cells expressing CTLA-4 and its MFI value in RRMS patients compared to healthy controls (Figure 3, Table S1). 

Among RRMS patients with a significantly increased percentage of CD8+ Tregs with GITR expression, there was a significantly lower GITR MFI value among both CD4+ T cell populations (CD4+CD25+ and CD4+CD25+FoxP3+), compared to healthy controls (Figure 3, Table S1). 

In contrast, there were no changes in the percentage of PD-1 positive cells; however, expression (MFI—mean fluorescence intensity) of PD-1 was significantly lower within all CD4+ and CD8+ T cell populations in RRMS (Figure 3, Table S1). Generalized linear modeling with a log-normal distribution was used to simultaneously assess the effects of EDSS. The analysis showed no significant effects of gender and age. There were no differences in PD-1-positive cells and MFI associated with EDSS in RRMS patients. 

The percentage of FoxP3+ and Helios+ cells within CD4+ and CD8+ T cell populations was increased in RRMS patients, though a significantly lower Helios MFI value was observed for RRMS patients compared to healthy controls. Expression of CD28 in RRMS patients was significantly lower only among both CD4+ T cell populations (CD4+CD25+ and CD4+CD25+FoxP3+). There were no differences in the frequency of CD62L+ and CD103+ cells in RRMS patients versus healthy controls. The expression of CD25 was markedly higher in CD8+CD122+ and CD8+CD122+Helios+ cells (Table S1). 

### 2.4. Profiles of Cytokines in Serum from RRMS Patients

**Figure 4:**
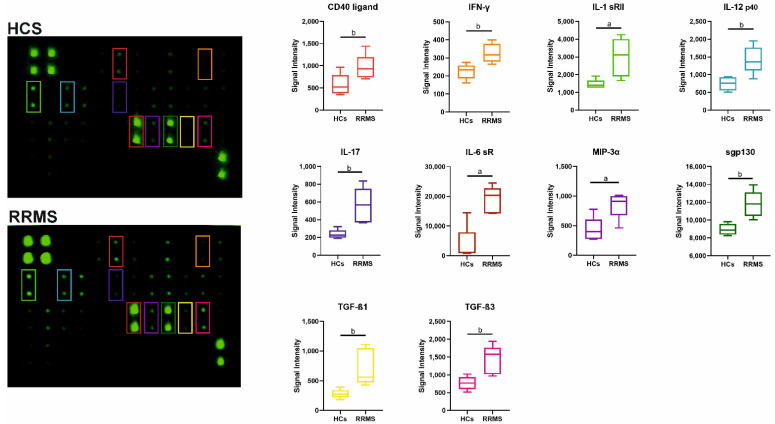
Identification of serum Th1/Th2/Th17 cytokines by antibody arrays. The serum cytokines differentially expressed between patients at pre-treatment (RRMS) and healthy controls (HC). Colored boxes indicate the locations of ten significantly different proteins on the arrays, and different colored boxes represent respective cytokines. Only those cytokines whose expression level (Relative Signal Intensity) differed in RRMS patients compared to the control are shown. Horizontal lines indicate significantly different groups ^a^ Mann–Whitney U-test or ^b^ *t*-Student test. RRMS *n* = 5, HC *n* = 5.

The Th1/Th2/Th17 Antibody Array assessed 34 biomarkers. Ten cytokines (CD40, IFN-*γ*, IL-17, IL-17F, TGF*β*-1, TGF*β*-3, IL-1 SRII, IL-12 p40, IL-6sR, sgp-130) had significantly increased values in RRMS patients when compared to healthy controls. Their profiles are shown in Figure 4. Detailed data for all cytokines are presented in Supplementary Table S2. To validate the microarray analysis, ELISA was performed for transforming growth factor beta-1 (TGF*β*-1), IL-10 and Interleukin-6 soluble receptor (IL-6sR) in serum. TGF*β*-1, but not IL-10 and IL-6sR, was significantly different in RRMS patients, a result identical to the microarray.

## 3. Discussion

This study has determined the percentages of regulatory T cell subpopulations in the peripheral blood and examined the associations with disease, EDSS, gender and age. Most cells were CD8+CD122+Helios+ or CD4+CD25+FoxP3+. Individuals with RRMS had more CD8+CD25+FoxP3+ cells but fewer CD8+CD122+ cells and fewer CD4+CD25+ cells than controls. Females had more CD8+CD122+Helios+ cells and more CD8+CD25+FoxP3+ cells than males. Older individuals had more CD4+CD25+ cells.

Ideally, each patient would have one or more controls matched for age, sex and all other variables affecting susceptibility or disease progression but this is seldom possible. Especially now, during the COVID-19 pandemic, few people, especially women, choose to donate blood. The use of generalized linear modeling is an alternative that allows valid conclusions to be drawn even when the study population does not contain equal numbers of both sexes in the control and disease groups. In addition, the variation among individuals was seldom normally distributed and generalized linear modeling can analyze both normal and non-normal distributions. 

The increased number of CD8+CD25+FoxP3+ cells in RRMS patients is consistent with a recent meta-analysis and suggests a role for CD8+ T cells in MS [14]. In contrast to effector T cells, Tregs strongly depend on a stable, anergic and suppressive phenotype to maintain immune homeostasis [15]. Several immunosuppressive markers such as CD25, CTLA-4, PD-1, GITR and transcription factors FoxP3 and Helios have been observed on both CD8+ and CD4+ T cells [16]. Here, we demonstrated that CD4+CD25+ and CD8+CD122+ T cells in the blood are decreased in RRMS patients compared to healthy controls. The cells expressed more FoxP3 but less Helios (Table S1). Helios stabilizes the suppressive phenotype and the activity of both CD4+ Tregs and CD8+ Tregs, by activating the STAT5 signaling pathway [17]. The results suggest that these Tregs have an unstable phenotype and that they migrate to inflammatory CNS lesions.

There was reduced expression of the suppressive marker PD-1 but no changes in CTLA-4 levels on CD8+ T cells and CD4+ T cells in RRMS patients. EDSS did not correlate with changes in PD-1 expression. The reduced expression of PD-1 in Tregs may explain the lack of suppressive activity of Tregs in RRMS [1]. Reduced expression of PD-1 leads to decreased suppressive activity of Tregs in vivo [18]. PD-1 is a monomeric transmembrane glycoprotein that shares homology with the B7/CD28 family of T cell signaling molecules [19,20]. PD-1 interacts with its ligands PD-L1 and/or PD-L2 to provide a negative regulatory signal to CD4+ and CD8+ T cells that results ultimately in a phenotype termed T cell exhaustion [21]. This may be an important pathogenic factor in RRMS, given that PD-1 is a strong inhibitor of T cell activity. The predominance of PD-1 interactions in the mechanism of peripheral tolerance is consistent with the expression of its ligands in most tissues [22]. PD-1 and CTLA-4 gene expression are significantly downregulated in PBMCs of RRMS patients compared with healthy controls [23,24]. Myelin basic protein (MBP)-stimulated T cells demonstrated a significantly increased frequency of PD-1-positive cells in stable MS compared to acute MS [25]. In murine models, blocking the PD-1 pathway via genetic knockdown or through the administration of blocking antibodies increases the risk for developing autoimmune dilated cardiomyopathy and experimental autoimmune encephalomyelitis [26]. Reduction in PD-1 expression on T cells in RRMS may result in a lack of PI-3K dephosphorylation, resulting in a lack of Akt kinase inhibition and an increase in the level of pro-inflammatory cytokines [27].

GITR+ CD8 Tregs were increased in RRMS but the expression of GITR was not upregulated in these cells. However, CD8+ T cells express GITR after activation [28]. GITR may abrogate the suppressive function of Tregs [29], and support the immunological tolerance of the self.

Ten cytokines (CD40, IFN-*γ*, IL-17, IL-17F, TGF*β*-1, TGF*β*-3, IL-1SRII, IL-12 p40, IL-6sR and sgp-130) were elevated in RRMS patients compared to healthy controls. The increased concentration of IFN-*γ*, IL-12, IL-17, TGF*β* -1 and TGF*β*-3 in serum is accompanied by the low expression of PD-1 on CD4+ and CD8+ T cells in the blood of RRMS patients. IL-12 enhances the ability of CD8+ T cells to modulate autoantigen-specific effector responses [12] and stimulates the production of IFN-*γ* and IL-17 by Th1 and Th17 cells [30]. TGF*β*-1 is one of the most important anti-inflammatory cytokines and plays an important role in the development of Th17 proinflammatory cells and Tregs. However, in MS, SMAD7 negatively regulates the TGF*β* signaling pathway. Its decreased expression can lead to increased expression of TGF*β*-1 and the promotion of Th17 polarization [31]. Several recent lines of evidence suggest a role for TGF*β*-3 in the pathogenesis of autoimmune diseases [32]. The significantly elevated level of sgp130 and IL-6sR in patients with RRMS indicates a role for the IL-6 trans-signaling pathway in the pathophysiology of RRMS. The trans-signaling mechanism is critical in the switch from the initial recruitment of neutrophils during acute inflammation to the recruitment of lymphocytes in the chronic phase [33]. However, further study will be necessary to define the relationship between IL-6 trans-signaling and PD-1 expression on Tregs in RRMS.

## 4. Materials and Methods

### 4.1. Study Design and Human Subjects

Blood samples from MS patients were provided by the Military Institute of Aviation Medicine in Warsaw. Informed, written consent was obtained from all donors. All uses of human material were approved by the Federal Office of Public Health (authorization No. 17/2019). The relapsing–remitting MS (RRMS) patient group included patients with the first attack of the type seen in MS. All patients met the 2010 Revised McDonald Criteria for a diagnosis of MS. The RRMS clinical form was determined according to the classification of Lublin and Reingold [34]. The clinical disability was evaluated using the Kurtzke Expanded Disability Status Scale (EDSS) [35] at the time of patient enrollment. All patients were categorized as mild (0.0–3.0) [36]. The lesion location (brain, spinal cord and optic nerve) and the results of Gadolinium (Gd) contrast-enhancing lesions were obtained from the MRI scan. Disease activity was identified as a GD-enhancing lesion on MRI. RRMS patients were naïve to MS disease-modifying therapy. Patients with RRMS did not have a history of malignancies and had no acute or chronic infections. Blood samples were collected at clinical onset of disease before starting treatment. 

The control group consisted of unrelated healthy blood donors who were from the same geographical area as the patients. Blood samples were obtained from the Regional Blood Donation and Blood Treatment Center in Warsaw. A total of 35 healthy controls (HC) and 15 relapsing–remitting MS (RRMS) patients were included in this study between September 2019 and December 2020. Demographic and clinical characteristics are presented in Table 2.

### 4.2. Collection of Serum Samples

Blood samples were collected by antecubital venipuncture. Blood samples were centrifuged at 3000 rpm for 15 min and then at 6000 rpm for 15 min. Serum was aliquoted and stored at − 80°C until analysis. 

### 4.3. Peripheral Blood Mononuclear Cell (PBMC) Isolation

PBMCs from RRMS patients and healthy donors were isolated by density centrifugation as described previously [37]. The total PBMC number and viability were quantified using the Muse Count and Viability Kit (Merck-Millipore, Billerica, MA, USA) followed by a Muse Cell Analyzer (Merck-Millipore), in accordance with the manufacturer’s instructions. The viability of isolated PBMCs was >97%.

### 4.4. Flow Cytometry Analysis

T regulatory cells were characterized by flow cytometry according to a protocol described previously [37]. The following fluorochrome-conjugated monoclonal antibodies were used according to the manufacturers’ protocols: CD3-eFluor506 (clone UCHT1; eBioscience, San Diego, CA, USA), CD4-BUV496 (clone SK3; BD Biosciences, San Diego, CA, USA), CD8-APC-eFluor780 (clone RPA-T8; eBioscience), CD122-PerCP-eFluor710 (clone TU27; eBioscience), CD25-BV650 (clone M-A251, BD Biosciences), CD14-AF700 (clone: 61D3; eBioscience), CD16-AF700 (clone: eBioCB16; eBioscience), CD19-AF700 (clone: Hib19; eBioscience), CD152(CTLA-4)-PE (clone 14D3; eBioscience), CD357(GITR)-BV610 (clone 621; BioLegend, San Diego, CA, USA) or PE-eFluor610 (clone eBioAITR; eBioscience), CD279(PD-1)-FITC (clone MIH4; eBioscience), CD62L-PE-eFluor610 (clone MEL-14; eBioscience), CD103-SB780 (clone B-LY7; eBioscience), CD28-eFluor506 (clone CD28.2, eBioscience). Fixable Viability Dye (FVD) eFluor 455UV (eBioscience) was used as a vital dye to exclude dead cells. Intracellular analysis of FoxP3-APC (clone: PCH101; eBioscience) and Helios-PE-Cy7 (clone 22F6; eBioscience) was performed after fixation and permeabilization, using a FoxP3/Transcription Factor Staining Buffer Set (eBioscience) according to the manufacturer’s instructions. Cells were acquired on a 5-laser, 19-color CytoFLEX LX (Beckman Coulter, Inc, Brea, CA, USA) calibrated daily using CytoFLEX Daily QC Fluorospheres (Beckman Coulter). Compensation settings were conducted using single-stained cells or the VersaComp Antibody Capture Bead Kit (Beckman Coulter). Positive staining and gating strategy were determined by comparison to an unstained control and a fluorescence minus one (FMO) control. The lymphocytes were gated based on morphological parameters on a forward vs. side scatter (FSC-A/SSC-A) plot. Cell aggregates were removed from the analysis using a sequential gating strategy with forward scatter (FSC) and side scatter (SSC) parameters, as presented in Figure 1. Data were analyzed using Kaluza Analysis Software version 2.1 (Beckman Coulter). The results are shown as the percentage of positively labeled cells, and the mean fluorescence intensity (MFI) was calculated by CytoFLEX LX. 

### 4.5. Cytokine Profiles

The human Th1/Th2/Th17 Antibody Array (ab169809, Abcam) was used to measure the concentration of 34 cytokines (CD30, CD40 ligand, CD40, G-CSF, GITR, GM-CSF, IFN-*γ*, IL-1-*β*, IL-1 sRI, IL-1 sRII, IL-2, IL-4, IL-5, IL-6, IL-6sR, IL-10, IL-12 p40, IL-12 p70, IL-13, IL-17, IL-17F, IL-17R, IL-21, IL-21R, IL-22, IL-23 (p19), IL-28A, MIP-3*α*, spg130, TGF-*β*1, TGF-*β*3, TNF-*α*, TNF-*β*, TRANCE) in serum (diluted 1/10) according to the manufacturer’s instructions. The exposure time was 5 min and examination was completed within 20 min as chemiluminescence signals will fade over time. The membranes were scanned with Syngene G-Box and the signal values were analyzed with Image J software. Signals were normalized using internal positive and negative controls included on the array.

### 4.6. ELISA

Serum TGF-*β*1, IL-10, IL-6sR were measured with the Human/Mouse TGF-beta1 Uncoated ELISA Kit (Invitrogen, Vienna, Austria), Human IL-10 Uncoated ELISA Kit (Invitrogen), DuoSet Human IL-6R*α* (R&D Systems, Minneapolis, MN, USA), respectively, according to the manufacturers’ instructions. The serum dilution factor 1/6 was chosen after titration. The readings were taken in triplicate, and the optical densities (OD) read at 450 nm with a Synergy™ H1 Microplate Reader (BioTek, Winooski, VT, USA) were compared with the standard curves prepared using recombinant proteins. 

### 4.7. Statistical Analyses

Fisher’s exact test was used to determine the significance of demographic factors. Proc Univariate (SAS on demand for academics, SAS Institute Cary, N. Carolina) was used to examine the distribution of each T cell subclass, and empirical distribution function tests (Anderson–Darling, Cramer von Mises and Kolmogorov–Smirnov tests) were used to test the fit of different distributions. The gamma distribution provided a better description of CD8+CD122+, CD8+CD25+, CD8+CD25+FoxP3+ and CD4+CD25+ cells than the log-normal or normal distributions. The log-normal distribution provided a better description of the distribution of CD4+CD25+FoxP3+ cells and the normal distribution provided a better fit for the distribution of CD8+CD122+Helios+ cells. Generalized linear modeling in SAS (SAS Institute Cary, N. Carolina) with the glimmix procedure was used to examine the effect of disease, age and sex on each subpopulation of cells. Antibody microarray data and ELISA data were analyzed with GraphPad Prism version 6.00 (Graphpad Software Inc., San Diego, CA, USA). Student’s *t* test or the Mann–Whitney rank sum test was used, according to the distribution of the data. In addition, fold change (FC) was calculated and presented, and the values given to indicate the relative expression levels of detected cytokines. For all tests in R, SAS and GraphPad Prism, differences were considered significant when *p* ≤ 0.05. The detailed description of the statistical analyses can be found in S3 Supplemental Material for Statistical Analyses.

## 5. Conclusions

RRMS individuals had fewer CD4+CD25+ and CD8+CD122+ T cells and more CD8+CD25+FoxP3+ cells. However, all the Tregs—CD8+CD122+Helios+, CD8+CD25+FoxP3+, CD4+CD25+FoxP3+—expressed less PD-1 and an unchanged level of CTLA-4. These findings provide new insights into the composition of CD4+ and CD8+ T cells in RRMS patients and may contribute to the development of T-cell-based adoptive immunotherapy for MS.

## Figures and Tables

**Table 1:** Model. Factors that partly explained the variance; *p* ≤ 0.05. EDSS parameter for RRMS group only; + positive correlation, − negative correlation.

Population	Age (Increase)	Gender (Female)	RRMS	EDSS (Increase)
CD8+ CD8+CD122+			−	
CD8+CD122+Helios+		+		
CD8+CD25+ CD8+CD25+FoxP3+		+	+	
CD4+				
CD4+CD25+	+		−	−
CD4+CD25+FoxP3+				

**Table 2:** Gender and age of healthy subjects and patients with RRMS characteristics. Baseline clinical characteristics.

Characteristics	RRMS Patients (*n* = 15)	Healthy Controls (*n* = 35)	*p*-Value
Gender. *n* (%)			
Female	13 (86.7)	12 (33.3)	0.0083
Male	2 (13.3)	24 (66.7)	
Age. years			
Median	30	38.5	0.0004
Range	19.0–47.0	19.0–57.0	
Treatment	No	No	-
EDSS score			
Median	1.5	-	-
Range	(1.0–3.0)	-	-

Fisher’s exact test (*p* ≤ 0.05). RRMS, relapsing–remitting multiple sclerosis; EDSS, Expended Disability Status Scale.

## Data Availability

The date presented in this study are available on request from the corresponding author.

## Supplementary Materials

The following supporting information can be downloaded at: https://www.mdpi.com/article/10.3390/ijms23063185/s1.
