# Supplementary material for: Reduced Expression of PD-1 in Circulating CD4+ and CD8+ Tregs Is an Early Feature of RRMS
Source: Int J Mol Sci. 2022 Mar 16;23(6):3185. doi: 10.3390/ijms23063185 (PMC8954486; doi:10.3390/ijms23063185)
Supplement: Supplementary file 1 [file ijms-23-03185-s001.zip › ijms-1598838-supplementary.v2/S3. Supplemental Material for Statistical Analyses (2).pdf]

S3. Supplemental Material for Statistical Analyses.

The age in years was fitted as a covariate while gender (male or female) and disease status (healthy or RRMS) were fitted as fixed effects. A separate model was used for each population of cells and the gamma, lognormal or normal distributions were fitted as appropriate. The gamma distribution was fitted with a log link. The normal and lognormal distributions were fitted with an identity link. Type III tests were used because these tests fit all effects simultaneously and significance is not influenced by the order the effects are fitted in the model. If there was a significant difference between patients and controls. the influence of disease severity was tested in patients only by fitting disease index score as a covariate. As in the other models, gender was fitted as a fixed effect while age was fitted as covariate. Two tailed tests were used.

Welch’s unequal variances two-tailed t-test was used to test for differences between the mean percentage of cells of the given marker and the MFI of the given marker. These analyses were performed with R version 4.0.3 (R Core Team (2013). R: A language and environment for statistical computing. R Foundation for Statistical Computing, Vienna, Austria. URL http://www.R-project.org/).
