# Supplementary material for: Reduced Expression of PD-1 in Circulating CD4+ and CD8+ Tregs Is an Early Feature of RRMS
Source: Int J Mol Sci. 2022 Mar 16;23(6):3185. doi: 10.3390/ijms23063185 (PMC8954486; doi:10.3390/ijms23063185)
Supplement: Supplementary file 1 [file ijms-23-03185-s001.zip › ijms-1598838-supplementary.v2/Tab.S1.pdf]

Table S1. Differential expression of activation and **suppressive markers on CD4+ and CD8+ T cells populations** . Results are the mean of the percentage of positively labelled T cells and mean fluorescence intensity (MFI). *compared to Healthy Controls ($p \leqslant 0.05$);

**Marker**	**Population**	**Healthy controls**		**RRMS patients**	
		**%**	**MFI**	**%**	**MFI**
**CTLA-4**	$CD4^+CD25^+$	0.15	1960	0.34	1944
	$CD4^+CD25^+FoxP3^+$	0.18	2131	0.36	1723
	$CD8^+CD122^+$	0.26	1859	0.77*	1495*
	$CD8^+CD122^+Helios^+$	0.19	1822	0.47	1490
	$CD8^+CD25^+$	1.31	1561	4.51	1477
	$CD8^+CD25^+FoxP3^+$	2.18	1529	5.82	1468
**GITR**	$CD4^+CD25^+$	7.62	4538	10.59	2787*
	$CD4^+CD25^+FoxP3^+$	4.82	3527	10.41	2720*
	$CD8^+CD122^+$	1.61	3110	5.35*	3307
	$CD8^+CD122^+Helios^+$	1.11	3163	4.47*	3231
	$CD8^+CD25^+$	9.01	3781	17.96*	3712
	$CD8^+CD25^+FoxP3^+$	11.29	4361	23.93*	3697
**PD-1**	$CD4^+CD25^+$	5.19	3923	3.44	3135*
	$CD4^+CD25^+FoxP3^+$	4.96	3874	3.24	3062*
	$CD8^+CD122^+$	8.14	4994	11.61	4085*
	$CD8^+CD122^+Helios^+$	8.11	4987	12.64	3755*
	$CD8^+CD25^+$	4.45	4222	3.55	2979*
	$CD8^+CD25^+FoxP3^+$	4.58	3413	5.46	3136*
**FoxP3**	$CD4^+CD25^+$	38.32	16705	42.88	18502*
	$CD8^+CD122^+$	2.38	13056	6.77*	22363
	$CD8^+CD25^+$	17.37	22139	22.31	36014*
**Helios**	$CD4^+CD25^+$	28.20	13594	32.23	10182*
	$CD4^+CD25^+FoxP3^+$	65.99	14342	68.15	10768*
	$CD8^+CD122^+$	51.13	11414	55.74	8769*
	$CD8^+CD25^+$	19.36	11017	31.08*	10214
	$CD8^+CD25^+FoxP3^+$	62.22	13699	67.49	11592
**CD28**	$CD4^+CD25^+$	97.76	10396	86.51*	9077*
	$CD4^+CD25^+FoxP3^+$	97.20	9310	83.07*	8724

	$CD8^+CD122^+$	35.62	8769	43.08	8892
	$CD8^+CD122^+Helios^+$	26.77	9207	37.07	9064
	$CD8^+CD25^+$	88.64	9786	81.37	10155
	$CD8^+CD25^+FoxP3^+$	95.67	10985	94.02	11079
**CD62L**	$CD4^+CD25^+$	87.47	21651	90.84	22478
	$CD4^+CD25^+FoxP3^+$	94.55	20416	94.88	21585
	$CD8^+CD122^+$	59.63	17410	57.88	12375*
	$CD8^+CD122^+Helios^+$	44.60	11579	50.68	10075
	$CD8^+CD25^+$	92.26	29539	95.07	31814
	$CD8^+CD25^+FoxP3^+$	94.45	25751	96.34*	30687
**CD103**	$CD4^+CD25^+$	4.48	3399	2.08*	3183
	$CD4^+CD25^+FoxP3^+$	1.90	2477	1.73	2614
	$CD8^+CD122^+$	0.99	2651	2.41*	3134*
	$CD8^+CD122^+Helios^+$	0.42	2760	0.96*	2872
	$CD8^+CD25^+$	12.75	3625	14.45	3763
	$CD8^+CD25^+FoxP3^+$	21.19	3727	14.64	3525
**CD25**	$CD8^+CD122^+$	1.30	2455	3.26*	3206*
	$CD8^+CD122^+Helios^+$	1.02	3049	2.77*	3502
