# Supplementary material for: Reduced Expression of PD-1 in Circulating CD4+ and CD8+ Tregs Is an Early Feature of RRMS
Source: Int J Mol Sci. 2022 Mar 16;23(6):3185. doi: 10.3390/ijms23063185 (PMC8954486; doi:10.3390/ijms23063185)
Supplement: Supplementary file 1 [file ijms-23-03185-s001.zip › ijms-1598838-supplementary.v2/Tab.S2.pdf]

Table S2. Protein microarray data (Relative Signal Intensity) detected cytokines from RRMS patients (RRMS) and healthy controls (HCs) serum.

**Cytokines**	**RRMS patients** (n=5)	**Healthy controls** (n=5)	**Fold change** (RRMS/HCs)	**P-value**
CD30	773.7	468.6	1.7	0.0674
CD40 Ligand	963.3	571.0	1.7	0.048 [b]
CD40	329.9	310.1	1.1	0.7861
G-CSF	31.0	38.2	0.8	0.5597
GITR	755.9	631.5	1.2	0.2739
GM-CSF	78.7	92.6	0.9	0.5675
IFN-γ	326.4	225.1	1.5	0.0099 [b]
IL-1 sRI	612.6	644.3	1.0	0.7741
IL-1 sRII	2984.1	1456.1	2.0	0.0159 [a]
IL-10	67.2	43.1	1.6	0.2516
IL-12 p40	1423.6	746.2	1.9	0.008 [b]
IL-12 p70	1059.2	742.7	1.4	0.1068

IL-13	298.9	184.2	1.6	0.1063
IL-17	561.6	236.9	2.4	0.0078 [b]
IL-17F	1095.7	194.1	5.6	0.0864
IL-17R	531.7	437.1	1.2	0.3172
IL-1β	946.2	741.2	1.3	0.1327
IL-2	436.9	404.2	1.1	0.5999
IL-21	862.1	500.2	1.7	0.1442
IL-21R	733.7	511.0	1.4	0.2011
IL-22	778.3	376.1	2.1	0.0541
IL-23 p19	2136.8	484.8	4.4	0.0513
IL-28A	559.5	415.9	1.3	0.0734
IL-4	557.0	417.0	1.3	0.1778
IL-5	89.9	122.5	0.7	0.1283
IL-6	101.9	61.8	1.6	0.2222
IL-6 sR	18901.3	3769.7	5.0	0.0317 [a]

MIP-3α	853.5	431.0	2.0	0.0159 [a]
sgp130	11789.0	8955.9	1.3	0.0043 [b]
TGF-β1	721.8	281.1	2.6	0.0139 [b]
TGF-β3	1429.9	769.0	1.9	0.0104 [b]
TNF-α	411.1	338.9	1.2	0.4163
TNF-β	626.6	260.7	2.4	0.1203
TRANCE	584.1	300.3	1.9	0.0600

[a]Mann Whitney test ($P \leqslant 0.05$). [b]t-Student test ($P \leqslant 0.05$). RRMS, relapsing-remitting multiple sclerosis; EDSS, Expended Disability Status Scale; HCs, healthy controls.
